# Temporal Features of the Differentiation between Self-Name and Religious Leader Name among Christians: An ERP Study

**DOI:** 10.3389/fpsyg.2017.02114

**Published:** 2018-01-25

**Authors:** Ruixue Xia, Ruijie Jin, Lin Yong, Shaodong Li, Shifeng Li, Aibao Zhou

**Affiliations:** ^1^School of Psychology, Northwest Normal University, Lanzhou, China; ^2^Key Laboratory of Behavioral and Mental Health of Gansu Province, Lanzhou, China

**Keywords:** self-name, religious leader, self-concept, P300, ERP

## Abstract

Existing neuroimaging studies have shown that religion, as a subjective culture, can influence self-referential processing. However, the time course of this impact remains unclear. The present study examined how Christians process their own names, the name of their religious leader (i.e., Jesus), and a famous person’s name (i.e., Yao Ming). Behavioral and EEG data were recorded while the participants performed a name-color judgment task for these three names. The behavioral data showed no significant differences in reaction time or accuracy among the names. However, the ERP data showed that the P200 and P300 amplitudes elicited by the self-name and religious leader name were larger than those elicited by the famous name. Furthermore, the self-name also elicited a larger P300 amplitude than the religious leader name did. These results suggested that both the self-name and the religious leader name were processed preferentially due to their important social value for the self as compared to a generally famous name. Importantly, the dissociation between the self-name and the religious leader name was observed at a high-order cognitive stage, which might be attributed to their different roles in one’s self-concept.

## Introduction

The exploration of the concept of ‘self’ can be traced back to ancient Greece. The significance of the ‘self’ has been central to the study of philosophy, psychology, and religion for centuries (Banaji and Prentice, 1994; Han et al., 2008). According to William James, “A man’s Self is the sum total of all that he CAN call his, not only his body and his psychic powers… All these things give him the same emotions” (James, 1890, p. 291) and construct the framework of one’s self. Thus, the self is the center of one’s universe of mind.

One’s self varies with the culture in which he/she is living (Markus and Kitayama, 1991; Heine, 2001). Considerable research comparing the self-referential processing of Western and Eastern Asian individuals has demonstrated that culture shapes the functional anatomy of the self-representation (Zhu and Zhang, 2002; Zhu et al., 2007). Researchers in this field also conceived the self-referential processing paradigm, which involves making trait judgments of the self, others, and the font of the words. Using this paradigm, researchers found that, unexpected recognition performance was better for self-trait judgments than for other-trait judgments (e.g., a famous person) in both Western and Eastern Asian participants. Furthermore, superior memory performance was found for mother-trait judgments in East Asian participants, but not in Western participants (Symons and Johnson, 1997; Zhu and Zhang, 2002). Neuroimaging studies have confirmed these behavioral findings. The ventral medial prefrontal cortex (VMPFC), which is a part of the cortical midline structures, is considered a neural basis for the self (Northoff and Bermpohl, 2004; Northoff et al., 2006 for review). The VMPFC was found to represent both the self and one’s mother for Eastern participants, whereas this region appeared to exclusively represent the self for Western participants (Zhu et al., 2007). Bicultural priming studies have provided further evidence for the influence of culture on the self-concept (Chiao et al., 2010). For example, participants’ neural differentiation between the self and the mother was increased in the American culture priming and decreased in the Chinese culture priming (Ng et al., 2010). These results suggested that culture might dynamically shape the self and its related mechanisms.

Recent studies have shown that religion, as a form of subjective culture, also shapes the neural mechanisms underlying self-referential processing (Han et al., 2008). For instance, Wu et al. (2010) found that there was no typical self-reference pattern (e.g., superiority of memory and activation in VMPFC) in Tibetan participants, due to Tibetan Buddhists’ pursuit of a minimal subjective sense of ‘I-ness’ (Lutz et al., 2007). Han et al. (2008, 2009) scanned Chinese Christians and Chinese Buddhists and found that, for the believers in both groups, self-referential processing was associated with increased activity in the dorsal medial prefrontal cortex (DMPFC), but not in the VMPFC. The DMPFC is associated with self-related reappraisal (Northoff et al., 2006) as well as inferences and evaluations of others’ mental states (Gallagher et al., 2000; Grèzes et al., 2004). These findings therefore might be explained by the fact that, because Christian doctrine urges the pursuit of self-transcendence (i.e., to deny oneself in order to live a spiritual life as dictated by Jesus; Ching, 1984; Mcdaniel, 1987), the believers might judge themselves from God’s perspective.

Indeed, Ge et al. (2009) showed that the trait judgment of religious leaders and the self had similar cognitive strategies and functional connectivity between the medial prefrontal cortex (MPFC) and posterior parietal cortex (PPC)/precuneus. For Christians, both self-judgments and judgments of their religious leader (Jesus) rely on a semantic trait summary that involves a minimum degree of episodic memory retrieval. More specifically, the functional connectivity between MPFC and precuneus/PPC, an index of episodic memory retrieval, was found to be decreased in self- and religious-leader judgments for Christians, but only in self-judgments for non-believers (Ge et al., 2009). Self-judgment is achieved by accessing a database of summary traits in semantic memory that has been abstracted from people’s experiences of trait-relevant behaviors (Klein et al., 1992, 2002). Trait judgments of specific others might also be accomplished by accessing trait knowledge in semantic memory if one has sufficient experience with that other, such as in the case of one’s mother (Klein et al., 1992, 2002). Empirical evidence has shown that processing mothers’ names or trait judgments referencing the mother elicited comparable behavioral performances (Zhu and Zhang, 2002); a comparable magnitude of VMPFC activity (Zhu et al., 2007; Wuyun et al., 2014); and a comparable magnitude of P200, N250, and P300 amplitudes (Tacikowski et al., 2014; Shi, 2016) as processing the self. For the believers of Christianity, Jesus has a special position as the creator and leader of Christianity (John 1:2). Daily Bible readings and religious practices might also help Christians accumulate considerable knowledge of Jesus (Ge et al., 2009). Therefore, Christians might form a trait summary of Him, resulting in a similar cognitive strategy and brain functional connectivity as for the self.

However, for the believers, there might also be some potential processing differences between the self and religious leaders. For example, Han et al. (2008, 2009) did not find any memory advantage in the religious leader judgments condition, but did find one in the self-condition, for both Christians and Buddhists. Han et al. (2008) also revealed that the activation of the right inferior parietal cortex, a region engaged in self-other distinction during self-recognition (Uddin et al., 2006), showed significant differences between judgments of the self and religious leader. Meanwhile, in Christianity, the believers view Jesus as “the image of the invisible God” (Colossians 1:15), and cannot physically meet or contact Him like they can their mothers. Thus, it is still unclear whether the processing of the religious leader and self would show similar patterns.

In the present study, we examined how 21 Christians process the self and their religious leader by recording EEGs as they performed a name-color judgment task for their own name, the name of their religious leader (Jesus), and a famous name (Yao Ming). A person’s name is a unique and inherent part of his or her concept of self (Perrin et al., 2005). The self-name can capture one’s attention in an automatic manner and is processed deeply because of its important value for the self (Moray, 1959; Alexopoulos et al., 2012). As trait judgments, specific others’ names can achieve such attentional superiority when those others are close or important to the self (Tacikowski et al., 2011a; Wuyun et al., 2014). As compared to previous research using explicit tasks, such as the trait judgment task (Zhu et al., 2007) or name familiarity judgment task (Tacikowski et al., 2014), the present study employed an implicit task—the name-color judgment task. This is because such implicit tasks are unrelated to the processing of the self and therefore would prevent potential confounding variables (e.g., judgment, evaluation, and categorization) from affecting the results (Wuyun et al., 2014). Therefore, the nature of the self can be better investigated. We predict that no significant behavioral differences would appear between the self-name, religious leader name, and famous name processing conditions because the same color judgment task would be used in all conditions.

As for the electrophysiological data, we chose the P200, N250, and P300 as indicators because these components are well-documented to distinguish the self from others at different cognitive processing stages. For example, self-referential information (participant’s name, date of birth, and hometown) elicits a large P200 and P300 (Hu et al., 2011; Tacikowski et al., 2014), and self-relevant possessive pronouns elicit a large P300 (Zhou et al., 2010). Furthermore, self-name, self-face, and self-relevant object recognition evoked a larger N250 (Tanaka et al., 2006; Miyakoshi et al., 2007, 2008; Zhao et al., 2009) than did other-referential processing. The P200, which occurs within 200 ms of stimulus onset, is indicative of rapid detection of typical stimulus features (Thorpe et al., 1996; Yuan et al., 2007) and recruitment of attention resources (Thorpe et al., 1996; Chen et al., 2008), and is maximized over the frontal-central scalp sites. The P200 usually indicates the perceptual processing of a stimulus’ physical attributes, which is automatic and unconscious (Hillyard and Anllo-Vento, 1998; Chen et al., 2008). The N250 occurs around 250 ms after stimulus onset, and is related to matching the input stimulus to stored knowledge in long-term memory (Miyakoshi et al., 2007). Finally, the P300 occurs around 300 ms after stimulus onset, and reflects the categorization and evaluation of stimuli in a higher-order cognitive stage (Johnson, 1988). It is usually distributed over the central-parietal scalp sites. The amplitude of the P300 correlates with the amount of attentional resources required for processing stimuli (Polich, 2007; Tacikowski et al., 2014; Demeter et al., 2016). All of this evidence illustrates that self-relevant stimuli receive preferential processing as compared to non-self-relevant stimuli due to their important social/adaptive value (Tacikowski and Nowicka, 2010; Tacikowski et al., 2011a, 2013). Compared with existing functional magnetic resonance imaging (fMRI) studies, the high temporal resolution of event-related potentials (ERPs) can help us explore the real time course of the mechanism of self-processing. Based on previous findings (Tacikowski et al., 2014; Shi, 2016), we expect that the self-name and religious leader name would be processed preferentially at the early perception stage because both of them are important to one’s self. Furthermore, the self-name and religious leader name would be differentiated at the late cognitive processing stages because of their different roles in one’s self.

## Materials and Methods

### Participants

Twenty-one Chinese Christians (10 females, 20–26 years old, mean age: 22.52 ± 1.91) who were recruited through community advertisements to complete a web-based screening participated in this study. All participants had been members of a community in Anning district of Lanzhou city, the People’s Republic of China for more than 3 years. All participants reported attending Sunday worship (about 2 h) and a communion (about 3 h) every week, and other religious activities, such as praying and singing. No participants had changed their names and all were right-handed, had normal or corrected-to-normal vision, and were without any obvious history of neurological or psychiatric problems. All participants were paid for their participation. The scientific and research Ethics Committee of the School of Psychology Northwest Normal University approved the experimental protocol and informed consents were obtained from all the subjects prior to the study.

### Stimulus and Procedure

All participants seated in a soundproof ERP laboratory. Three types of stimuli were employed in the experimental task: the participants’ own name, their religious leader name (Jesus), and the famous name (e.g., Yao Ming). Before the experiment, thirty college students (16 females, 22–27 years old, mean age: 23.93 ± 1.30) were recruited to evaluate the familiarity of six Chinese athletes, such as Yao Ming, Liu Xiang, etc. Every name’s familiarity was rated on a nine-point Likert scale ranging from 0 (not at all) to 9 (extremely familiar). The result showed Yao Ming was the most familiar athletes to the participants (*M* = 7.5, *SD* = 1.82). Each name was presented visually 60 times (half in blue and half in green), and the visual angle of the stimuli was 2.6° × 1.5°. In the experiment, participants were asked to judge the color of the names by pressing a button. Half were instructed to press ‘1’ for ‘blue’ and ‘4’ for ‘green,’ and vice versa. Trials were randomized across conditions; all 180 stimuli were evenly distributed into the two blocks, and one block lasted about 5 min on average with a brief break in between. At the start of each trial, a small black cross which prompted the participants to stare at the screen appeared on a gray screen for 200 ms, which was followed by a blank screen for 200–400 ms. Next, one of the names was displayed for 800 ms, and the participants were required to respond during that time. After the visual presentation of stimuli, a blank screen was presented for 1400 ms.

### Data Analysis

#### Behavioral Data

Response times and accuracy rates were recorded for each name type separately. Responses were scored as correct if the appropriate key was pressed between 300 and 2000 ms after the stimulus onset, and reaction times were only analyzed for correct trials. Both reaction times and accuracy rates were analyzed using repeated measures ANOVA with name type as a within-group variable. The Greenhouse–Geisser correction was applied wherever necessary, and Bonferroni correction was used to control for Type I error for multiple comparisons.

#### ERP Recordings and Analysis

EEG data were continuously recorded on line with a sampling rate of 250 Hz from the 256-channel HydroCel Geodesic Sensor Net (Electrical Geodesics, Inc., Eugene, OR, United States) referenced to the Cz (impedance < 50 kΩ) on line. The data were analyzed offline using NetStation 4.5.7 analysis software. The raw EEG data were digitally filtered with a 0.1–45 Hz bandpass filter, and segmented to epochs of 1000 ms after name onset with a 200 ms pre-stimulus baseline. For each trial, channels were marked as artifacts if signal variation exceeded ± 100 μV. Trials with artifacts in more than 10 channels were excluded from the analysis. For trials with artifacts in less than 10 channels, an algorithm that derived values from neighboring channels using a spherical spline interpolation was used to replace bad channels. Trials were excluded if signal variation of vertical and horizontal electrooculograms exceeded ±70 and ±22.5 μV, respectively. On average, for each participant, we retained more than 40 trials per condition after artifact detection. Following trial rejection, ERPs were transformed using average reference and then corrected using a 200 ms baseline.

Separate ERPs were formed for each of the experimental conditions, each of the subjects and each of the electrode sites. According to the scalp distribution of each ERP component in the present study, and based on the previous relevant studies (Csépe et al., 2003; Tacikowski and Nowicka, 2010), the mean amplitudes of the P200 (60–216 ms), N250 (140–300 ms), and P300 (240–600 ms) was calculated and submitted to a 3 (name type: self-name, religious leader name, famous name) × 6 (electrode: P200: F3, F4, Fz, C3, C4, Cz; N250: F3, F4, Fz, C3, C4, Cz; P300: C3, C4, Cz, P3, P4, Pz) two-way repeated measures ANOVA which was calculated by SPSS21.0. The electrodes were selected according to the international 10–20 system. The Greenhouse–Geisser correction was applied when necessary and Bonferroni correction was used for multiple comparisons.

## Results

### Behavioral Results

In terms of the behavioral data, the ANOVA did not show any significant main effect or interaction effect for reaction time and response accuracy (*F*s < 2.039, *p*s > 0.05) (see **Table 1**).

**Table 1:** The mean scores and standard deviance of Response accuracy (%) and Reaction time (ms) in self-name condition, religious leader name, and famous name condition.

	Self-name	Religious leader name	Famous name	*F*(2,40)	η^2^
	*M (SD)*	*M (SD)*	*M (SD)*		
Response accuracy (%)	96.19 (1.76)	95.94 (1.74)	95.32.90 (1.72)	2.039	0.093
Reaction time (ms)	458.11 (138.75)	458.30 (144.36)	452.23 (146.22)	0.309	0.015

### ERP Results

**FIGURE 1:**
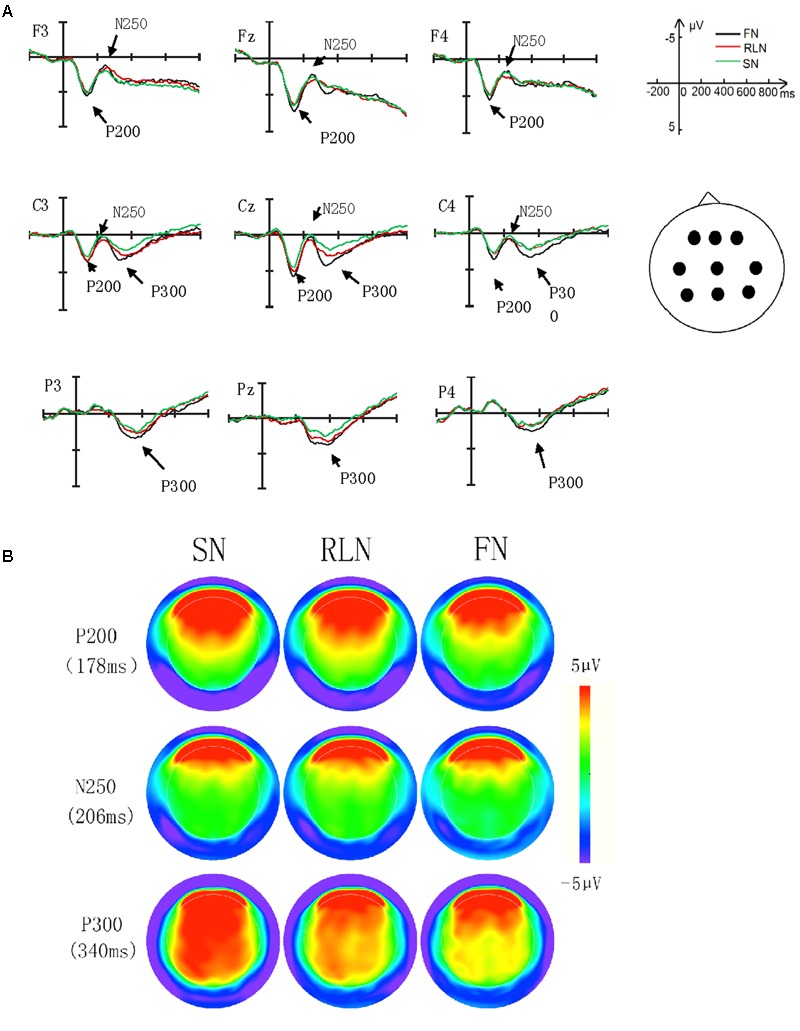
**(A)** Grand-average ERPs for self-name (SN), religious leader name (RLN), famous name (FN). ERPs are presented for electrode locations chosen for statistical analyses. **(B)** Topographical distributions of the ERP components-of-interest computed for all experimental conditions averaged together.

As shown in **Figure 1**, for the P200, the ANOVA showed a significant main effect of *name type* [*F*(2,40) = 6.78, *p* = 0.003, η^2^ = 0.25]. Bonferroni-corrected pairwise comparisons showed that the P200 amplitudes for the self-name and religious leader name were larger than for the famous name (*p* = 0.007, Cohen’s *d* = 1.27; and *p* = 0.015, Cohen’s *d* = 0.58, respectively), and there was no significant difference between the self-name and religious leader name (*p =* 0.07). The main effect of *electrode* was also significant [*F*(5,100) = 16.76, *p* < 0.001, η^2^ = 0.46]; overall, the amplitudes elicited in the frontal region were larger than those in the central region. The interaction of *name type* × *electrode* was significant [*F*(10,200) = 2.65, *p* = 0.02, η^2^ = 0.12]. Simple effects analysis showed that the P200 amplitudes for the self-name and religious leader name were larger than that for the famous name at the Fz (*p* = 0.019), C3 (*p* = 0.01), Cz (*p* = 0.002), C4 (*p* = 0.026).

For N250, the main effect of *name type, electrode* and the interaction of *name type* × *electrode* were not significant (*F*s < 3.186, *ps* > 0.06) (see **Figure 1**).

For P300, a significant main effect of *name type* emerged [*F*(2,40) = 13.76, *p* < 0.001, η^2^ = 0.41]. Bonferroni-corrected pairwise comparisons revealed that P300 amplitudes for the self-name and religious leader name were larger than those for the famous name (*p* < 0.001, Cohen’s *d* = 2.02; and *p* = 0.017, Cohen’s *d* = 1.13, respectively). Meanwhile, the amplitude for the self-name was also larger than that for the religious leader name (*p* = 0.024, Cohen’s *d* = 0.93). The main effect of *electrode* was also significant [*F*(5,100) = 4.92, *p* < 0.001, η^2^ = 0.20]; overall, the amplitudes elicited in the parietal region were larger than those in the central region. The interaction of *name type* × *electrode* was not significant (*p* = 0.19) (data in **Figure 1** show).

## Discussion

In the present study, we used an implicit name-color judgment task to examine the neural activities of processing the self-name, a religious leader name, and a famous name among Christians. Behavioral data showed there were no significant differences among the three name types for both reaction times and accuracy rate. This might be attributed to our use of a color judgment task, in which task performance was unrelated to the names, as behavioral indicators might not be as sensitive to implicit tasks. This result is in line with Wuyun et al. (2014) finding that no significant differences were observed between processing of the self-name, the mother’s name, and a stranger’s name in an implicit name judgment task. However, Ma and Han (2009, 2010) used an implicit face orientation judgment task and found the self-referential processing advantage. The inconsistent findings between Ma and Han (2009, 2010) and the present study might be ascribed to the different stimuli. The first step in name processing is word form analysis, whereas the first step in face processing is structural encoding (Tacikowski et al., 2011b).

The present study showed that both the self-name and the religious leader name elicited larger P200 amplitudes than did the famous name. This result is consistent with previous findings related to the processing of the self-name and one’s mother’s name (Tacikowski et al., 2014). As mentioned above, the P200 is related to the detection of stimulus features in the perception stage (Thorpe et al., 1996; Yuan et al., 2008). Therefore, the present result indicated that both the self-name and religious leader name are more rapidly detected and captured attention more automatically in the early perceptual processing stage as compared to the famous name. The fact that the religious leader name had a similar level of processing preferentiality as the self-name can be explained by past research showing that religious faith influences the self-concept, mainly because the believers define themselves via their religious doctrine (e.g., the non-self orientation of Tibetan Buddhists, self-transcendence of Christians; Han et al., 2008; Wu et al., 2010). In this way, the definition of self for the believers might rely heavily on their religious doctrine, which is symbolized in their religious leader (Matthew 28:18; John 1:18; Hebrews 1:2,1:3). This finding was also partly compatible with the results of Ge et al. (2009), which showed that the believers used a semantic trait summary cognitive strategy and similar brain functional connectivity when they judged themselves’ and their religious leader’s traits. Combining the results of the present study and those of previous findings, we speculate religious faith and practices might cause the believers to incorporate their religious doctrine and religious leader into their self-concept, producing the results shown with the P200.

The findings concerning the P300, with the amplitudes for the self-name and religious leader name being larger than that for the famous name, added support for the P200 results. The P300 is related to attentional allocation of resources and evaluation of target stimuli in the late higher-order cognitive stage (for review, see Polich, 2007). Our results showed that the self-name and religious leader name were processed preferentially both at the perceptual stage and at the high-order cognitive evaluation stage. In the present study, the experimental task required the participants to identify the color of the self-name, religious leader name, and the famous name on a screen. The color sets and percentages of each color for each name were equivalent. Therefore, the processing advantages for the self-name and religious leader name, as indicated by the P200 and P300, cannot be attributed to differences in physical characteristics or task requirements across name type.

Interestingly, the self-name still elicited larger P300 amplitudes than did the religious leader name. This indicated that there is also some dissociation between processing of the self-name and the religious leader name. A person’s own name is an exclusive symbol of his/her identity and is closest to their core self (Shapiro et al., 1997). A large number of studies have supported that individuals show a self-name processing priority (Berlad and Pratt, 1995; Folmer and Yingling, 1997; Gray et al., 2004; Zhao et al., 2009). Even patients with dementia and those who are comatose can identify their own names (Fishback, 1977; Fischer et al., 2008). Processing the self-name might involve more elaborative cognitive processes and emotion (Tacikowski et al., 2014; Shi, 2016). Indeed, the occurrence of the self-name might signal potentially important information concerning the self (e.g., a warning, threat, praise) (Tacikowski et al., 2014), which might contribute to the larger P300 (Johnston et al., 1986; Conroy and Polich, 2007).

According to James, “There is the strongest lightness about the heart when one’s nothingness in a particular line is once accepted in good faith” (James, 1890, p. 301). Jesus is an interpreter of religious doctrines and plays a significant role as both moral supporter and behavior guide for the believers. The religious doctrines of Christianity teach the believers to keep peace in their minds and rely on God (Isaiah 30.15). All of these aspects of their religion might make the believers more relaxed, calm, and quiet. There is also evidence that religious and spiritual practices are associated with parasympathetic nervous system activities, which can lead to a reduction in heart rate, respiratory rate, blood pressure, and cortisol level (Sudsuang et al., 1991; Lumma et al., 2015). All these physiological responses are related to subjective feelings of relaxation and profound quiescence, which, in turn, might contribute to the smaller P300 amplitudes when processing the religious leader name than the self-name.

There were also some inconsistencies when comparing our findings with those of previous studies on mother’s name, which showed that there was no difference in processing of the self-name and mother’s name (Tacikowski et al., 2014; Shi, 2016). One possible reason for this inconsistency is that the shared experiences and deep emotional bond between the self and the mother might cause individuals to be more motivated in attending to their mother’s name, which might result in comparable P300 amplitudes between processing the self-name and the mother’s name (Tacikowski et al., 2014). Conversely, as mentioned above, the religious leader name might generate peaceful feelings for the believers, therefore leading to a smaller P300.

There are some limitations in the present study. First, all the participants were Chinese Christians. Since China is a non-Christian country, the external validity of the present study might be limited. Thus, further research is necessary to verify this result in different situations and cultural backgrounds. Second, since we did not set a mother-name condition, all the comparisons between findings for the mother name and religious leader name were referred to the previous relevant studies. Future studies should include both a mother name condition and a religious leader name condition to compare the processing of these two names more directly.

## Conclusion

We used ERPs to examine how Christians process their own names and the names of their religious leaders using an implicit name-color judgment task. The results showed that the self-name and the religious leader name were processed preferentially as compared with a famous name, both at the early perceptual processing stage and at the late higher-order evaluation stage (respectively indexed by the P200 and P300). These results suggested that the religious leader is closely related to a believer’s self (i.e., the believers might integrate their religious beliefs into their self-concept). Meanwhile, the self-name elicited a larger P300 in comparison to the religious leader name, suggesting a dissociation between these components of one’s self-concept. More studies are needed to investigate this association and dissociation within one’s self-concept.

## Author Contributions

AZ conceived this research. RX participated in writing the manuscript. RJ participated in performing research. LY participated in reviewing literatures. SdL participated in making graph and table. SfL participated in modifying the manuscript.

## Conflict of Interest Statement

The authors declare that the research was conducted in the absence of any commercial or financial relationships that could be construed as a potential conflict of interest.
